# Supplementary material for: Understanding Antibiotic Resistance as a Perceived Threat towards Dairy Cattle through Beliefs and Practices: A Survey-Based Study of Dairy Farmers
Source: Antibiotics (Basel). 2022 Jul 25;11(8):997. doi: 10.3390/antibiotics11080997 (PMC9330383; doi:10.3390/antibiotics11080997)
Supplement: Supplementary file 1 [file antibiotics-11-00997-s001.zip › antibiotics-1818131-supplementary.pdf]

Supplementary Material for

Understanding antibiotic resistance as a threat towards dairy cattle through beliefs and practices: a survey-based study of dairy farmers

Eleni Casseri, Ece Bulut, Sebastian Llanos Soto, Michelle Wemette, Alison Stout, Amelia Greiner Safi, Robert Lynch, Paolo Moroni, Renata Ivanek

Corresponding author:

Eleni Casseri

emc356@cornell.edu

Response and completion rate calculations

According to the guidelines of the American Association for Public Opinion Research (AAPOR), the response rate is defined as the number of respondents who provided a usable response (C+P) divided by all invitations sent (TS) (Table S1). The response rate in our study assumes that all invitees are eligible for the survey (since e-mails were sent to dairy farmers and managers only) and includes partial responses. The completion (participation) rate, on the other hand, is the proportion of the people who completed the survey (C) to those who started completing the survey (C+P).

We used two sample sizes to calculate the response rate: (1) sample size at individual level (1,390) and (2) sample size at farm level (890). Accordingly;

- Response rate for the total sample size at individual level = (C+P)/(TS) = (104+27)/1,390 = 9.4%
- Response rate for the total sample size at farm level = (C+P)/(TS) = (104+27)/890 = 14.7%
- Completion rate = C/(C+P) = 104/(104+27) = 79.4%

Table S1. Final survey sample status.

**Status[1]**	**Notation**	**Number**
Completed Survey	C	104
Partially completed survey[2]	P	27
Refusal[3]	R	NA[4]
Non-contact[5]	NC	1,259
Other[6]	O	NA[4]
**Total sample[7]**	**TS**	**1,390**

[1]Status of the respondents were categorized using the guidelines provided by the American Association for Public Opinion Research (AAPOR) [1].

[2]Partially completed with varying degrees. We included 14 of the 27 partially completed responses in our analyses since those respondents answered questions beyond the farm and demographic questions. The total number of responses to each question is provided in the manuscript (Tables 1 and 2).

[3]Respondents refused to participate in the study. By definition, refusals consist of respondents who were contacted but declined to do the survey. Since our survey was distributed via e-mail, we cannot know whether the invitee has declined. Therefore, this category is not applicable to our survey.

[4]NA: Not applicable

[5]Non-contact category is reserved for respondents when the invitee is considered eligible (since the survey was sent to farmers and managers only) but no response was obtained.

[6]Other cases represent instances in which the respondent is eligible and does not refuse, but the survey is not obtainable because of the following reasons defined by AAPOR and known by the researchers: death, physical or mental limitations, language problems, literacy problems or miscellaneous reasons (i.e., combination of other reasons or faked/invalidated cases, etc.). Since our survey was distributed via e-mail, we cannot know the reason for the lack of response. Therefore, this category is not applicable to our survey.

[7]The initial email list consisted of 1,401 email addresses, however, the Total Sample size reported in Table S1 is 1,390. The difference is due to our deduplication process, where we deleted duplicated e-mails.

Reference:

1. AAPOR (2016). Standard Definitions: Final Dispositions of Case Codes and Outcome Rates for Surveys. 9th edition. AAPOR. (Last accessed on May 16, 2022) https://www.aapor.org/AAPOR_Main/media/publications/Standard-Definitions20169theditionfinal.pdf

Table S2. The results of univariable analysis of the association between the binary version of the outcome of interest Q17 (Disagreed vs Agreed with the statement that antibiotic resistance due to antibiotic use in dairy farming may negatively impact the health of dairy cattle, referenced as RESISTANCE BELIEFS) and categorical explanatory variables.

**Question** [a]	**Level**	**Agree**		**Disagree**		**Odds ratio**	**95% confidence interval**		**p-value**	**Test** [b]
		Number	%	Number	%	Mean	lower	upper		
Q2	Conventional	49	0.88	42	0.91	1.00	NA	NA	NA	FE
	Organic	7	0.13	4	0.09	0.67	0.13	2.85	0.75	
Q3	Owner/co-owner	43	0.77	38	0.83	1.00	NA	NA	NA	Chi2
	Herd manager	13	0.23	8	0.17	0.70	0.26	1.86	0.47	
Q4 [c]	Over twenty years	24	0.43	16	0.35	1.00	NA	NA	NA	Chi2
	Between ten and twenty years	12	0.21	13	0.28	1.63	0.59	4.45	0.34	
	Between five and ten years	10	0.18	9	0.20	1.35	0.45	4.06	0.59	
	Up to five years	10	0.18	8	0.17	1.20	0.39	3.69	0.75	
Q8 [d]	Interested	48	0.86	41	0.89	1.00	NA	NA	NA	FE
	Uninterested	8	0.14	5	0.11	0.73	0.17	2.78	0.77	
Q10 [e]	Interested	49	0.88	44	0.96	1.00	NA	NA	NA	FE
	Uninterested	7	0.13	2	0.04	0.32	0.03	1.81	0.18	
Q12	Scientifically proven	22	0.40	25	0.54	1.00	NA	NA	NA	FE
	Allowed under organic certification	6	0.11	3	0.07	0.45	0.06	2.39	0.47	
	Cost of the product	12	0.22	6	0.13	0.45	0.12	1.55	0.18	
	Ease of administration	6	0.11	2	0.04	0.30	0.03	1.90	0.25	
	Milk withhold time	5	0.09	7	0.15	1.23	0.29	5.66	1.00	
	Other	4	0.07	3	0.07	0.67	0.09	4.41	0.70	
Q13	Herd veterinarian	36	0.65	30	0.65	1.00	NA	NA	NA	FE
	Cooperative Extension/Quality Milk Production Services	2	0.04	0	0.00	0.00	0.00	6.74	0.50	
	Dairy publications, such as Hoard's Dairyman	4	0.07	5	0.11	1.49	0.29	8.23	0.73	
	None, I am not interested in antibiotic alternatives	1	0.02	4	0.09	4.70	0.44	242.50	0.19	
	Other	5	0.09	3	0.07	0.72	0.10	4.07	0.73	
	Other dairy farmers	7	0.13	4	0.09	0.69	0.13	3.02	0.75	
Q14	Very helpful	21	0.38	22	0.48	1.00	NA	NA	NA	FE
	Somewhat helpful	24	0.43	15	0.33	0.60	0.23	1.57	0.27	
	Unsure	5	0.09	2	0.04	0.39	0.03	2.70	0.42	

	Not helpful	6	0.11	7	0.15	1.11	0.27	4.73	1.00	
Q15 [f]	Agree	37	0.76	26	0.62	1.00	NA	NA	NA	Chi2
	Disagree	12	0.24	16	0.38	1.90	0.77	4.67	0.16	
Q16	Financial incentives provided with your milk check	13	0.27	14	0.33	1.00	NA	NA	NA	FE
	Grants to upgrade facilities to reduce infection risk	10	0.21	8	0.19	0.75	0.19	2.88	0.76	
	None of the above	10	0.21	11	0.26	1.02	0.28	3.72	1.00	
	Other	6	0.13	3	0.07	0.47	0.06	2.80	0.45	
	Subsidized veterinary consulting/Quality Milk Production Services	9	0.19	6	0.14	0.63	0.14	2.64	0.53	
Q18 [g]	Agree	9	0.18	10	0.24	1.00	NA	NA	NA	Chi2
	Disagree	40	0.82	32	0.76	0.72	0.26	1.98	0.52	
Q5 [h]	Over 55	16	0.32	10	0.22	1.00	NA	NA	NA	Chi2
	18-34 years old	11	0.22	9	0.20	1.31	0.40	4.27	0.66	
	35-44 years old	15	0.30	17	0.38	1.81	0.63	5.19	0.27	
	45-54 years old	8	0.16	9	0.20	1.80	0.52	6.20	0.35	
Q6	Female	21	0.44	19	0.42	1.00	NA	NA	NA	Chi2
	Male	27	0.56	26	0.58	1.06	0.47	2.42	0.88	
Q7	College graduate (bachelor's degree)	28	0.56	28	0.62	1.00	NA	NA	NA	FE
	High school graduate or GED certificate	5	0.10	6	0.13	1.20	0.27	5.58	1.00	
	Post-graduate training or professional schooling after college	4	0.08	2	0.04	0.51	0.04	3.86	0.67	
	Trade school, associate's degree or some college	13	0.26	9	0.20	0.70	0.22	2.09	0.62	
Q37	More than 5 years	44	0.88	42	0.95	1.00	NA	NA	NA	FE
	Less than 5 years	6	0.12	2	0.05	0.35	0.03	2.12	0.28	

[a] The question (Q) number notations match those in the Qualtrics dataset.

[b] FE=Fisher's Exact Test; Chi2=Chi-squared Test

[c] In Q4, "Up to five years" included levels "Less than a year" and "Up to five years".

[d] In Q8, "Interested" included "I am already doing this fully", "I am already doing aspects of this", "I would be ready to do this in the near future", "I am interested in this but unable", while "Uninterested" included "I am not interested in this".

[e] In Q10, "Interested" includes "I am already doing/have done this fully", "I would be ready to do this in the near future", and "I am interested but unable", while "Uninterested" includes "I am not interested in this".

[f] In Q15, "Agree" includes "Strongly agree", "Somewhat agree", and "Agree", while "Disagree" includes "Neither agree nor disagree", "Somewhat disagree".

[g] In Q18, "Agree" includes "Strongly agree and "Agree", while "Disagree" includes "Strongly disagree" and "Disagree".

[h] In Q5, "18-34 years old" includes "18-24 years old" and "25-34 years old".

Table S3. Descriptive statistics and the results of univariable analysis of the association between the binary version of the outcome of interest in Q17 (Disagreed vs Agreed with the statement that antibiotic resistance due to antibiotic use in dairy farming may negatively impact the health of dairy cattle) and variables representing the numerical free choice survey questions as well as variables describing the calculated frequency of (i) clinical mastitis and (ii) injectable and (iii) intramammary antibiotic treatment doses per 100 lactating cows. [a,b]

	**Q17: Disagree**					**Q17: Agree**					**p-value [d]**
**Question No. [c]**	**Min**	**1st Quartile**	**Median**	**3rd Quartile**	**Max**	**Min**	**1st Quartile**	**Median**	**3rd Quartile**	**Max**	
Q23	39.0	200.0	472.5	1328.8	3700.0	5.0	80.0	265.0	1200.0	3200.0	0.14
Q31	0.0	12.0	35.0	88.5	300.0	0.0	5.0	25.0	85.0	300.0	0.21
Q24	0.0	3.0	6.0	14.5	173.0	0.0	1.0	6.0	20.0	125.0	0.83
Q25	0.0	0.0	5.0	17.0	250.0	0.0	0.0	0.5	12.8	108.0	0.15
Q26	0.0	2.8	10.0	26.3	150.0	0.0	0.0	5.0	37.5	150.0	0.43
(Q24/Q23)*100 [e]	0.0	0.9	1.3	2.7	12.6	0.0	0.8	2.2	3.7	8.0	0.35
(Q25/Q23)*100 [e]	0.0	0.0	1.3	4.0	26.7	0.0	0.0	0.1	2.6	24.0	0.12
(Q26/Q23)*100 [e]	0.0	0.5	1.9	4.3	24.6	0.0	0.0	1.8	5.7	28.6	0.60

[a] Question (Q32) “What was your average somatic cell count last month?” is not included due to discrepancies in how the numerical values were entered by the participants, and therefore statistical analysis was not possible.

[b] Participant with ID 105 was removed from this analysis because of an invalid response to Q24 (response entered was 0.02) and their response to Q23 was interpreted as a typo (response entered was 1).

[c] The question (Q) number notations match those in the Qualtrics dataset.

[d] Mann-Whitney U test.

[e] Frequencies were calculated by dividing the participant’s responses to Questions Q24, Q25 and Q26 in Table S3 by the number of lactating cows on their dairy farm (Q23).

Table S4. The results of univariable analysis of the association between the binary version of the outcome of interest Q8 (Uninterested vs Interested in adopting culture-based testing for treating mastitis, referenced as CULTURE-BASED TREATMENTS) and categorical explanatory variables.

**Question** [a]	**Level**	**Interested**		**Uninterested**		**Odds ratio**	**95% confidence interval**		**p-value**	**Test** [b]
		Number	%	Number	%	Mean	lower	upper		
Q2	Conventional	86	0.91	11	0.79	1.00	NA	NA	NA	FE
	Organic	8	0.09	3	0.21	2.89	0.43	14.62	0.15	
Q3	Owner/co-owner	73	0.78	11	0.79	1.00	NA	NA	NA	FE
	Herd manager	21	0.22	3	0.21	0.95	0.16	4.06	1.00	
Q4 [c]	Over twenty years	36	0.38	6	0.43	1.00	NA	NA	NA	FE
	Between ten and twenty years	20	0.21	6	0.43	1.78	0.42	7.68	0.51	
	Between five and ten years	19	0.20	0	0.00	0.00	0.00	1.81	0.16	
	Up to five years	19	0.20	2	0.14	0.64	0.06	4.02	0.71	
Q17 [d]	Agree	48	0.54	8	0.62	1.00	NA	NA	NA	FE
	Disagree	41	0.46	5	0.38	0.73	0.17	2.78	0.77	
Q10 [e]	Interested	85	0.91	12	0.86	1.00	NA	NA	NA	FE
	Uninterested	8	0.09	2	0.14	1.76	0.16	10.43	0.62	
Q12	Scientifically proven	48	0.53	1	0.07	1.00	NA	NA	NA	FE
	Allowed under organic certification	7	0.08	2	0.14	12.67	0.59	819.59	0.06	
	Cost of the product	16	0.18	3	0.21	8.65	0.64	480.48	0.06	
	Ease of administration	4	0.04	4	0.29	41.00	3.20	2361.90	0.00	
	Milk withhold time	12	0.13	1	0.07	3.88	0.05	319.44	0.38	
	Other	4	0.04	3	0.21	31.03	2.02	1895.03	0.00	
Q13	Herd veterinarian	61	0.67	8	0.57	1.00	NA	NA	NA	FE
	Cooperative Extension/Quality Milk Production Services	2	0.02	0	0.00	0.00	0.00	43.92	1.00	
	Dairy publications, such as Hoard's Dairyman	8	0.09	2	0.14	1.89	0.17	12.20	0.61	
	None, I am not interested in antibiotic alternatives	5	0.05	0	0.00	0.00	0.00	9.90	1.00	
	Other	7	0.08	1	0.07	1.09	0.02	10.56	1.00	
	Other dairy farmers	8	0.09	3	0.21	2.81	0.40	15.35	0.17	
Q14	Very helpful	41	0.45	4	0.29	1.00	NA	NA	NA	FE
	Somewhat helpful	35	0.38	6	0.43	1.75	0.38	9.12	0.51	

	Unsure	4	0.04	3	0.21	7.21	0.78	64.47	0.04	
	Not helpful	12	0.13	1	0.07	0.86	0.02	9.81	1.00	
Q15 [f]	Agree	58	0.72	5	0.50	1.00	NA	NA	NA	FE
	Disagree	23	0.28	5	0.50	2.49	0.52	11.97	0.27	
Q16	Financial incentives provided with your milk check	23	0.28	4	0.44	1.00	NA	NA	NA	FE
	Grants to upgrade facilities to reduce infection risk	17	0.21	1	0.11	0.35	0.01	3.92	0.63	
	None of the above	17	0.21	4	0.44	1.34	0.22	8.35	0.72	
	Other	9	0.11	0	0.00	0.00	0.00	4.66	0.55	
	Subsidized veterinary consulting/Quality Milk Production Services	15	0.19	0	0.00	0.00	0.00	2.67	0.28	
Q18 [g]	Agree	18	0.22	1	0.10	1.00	NA	NA	NA	FE
	Disagree	63	0.78	9	0.90	2.55	0.31	118.82	0.68	
Q5 [h]	Over 55	20	0.24	6	0.50	1.00	NA	NA	NA	FE
	18-34 years old	19	0.23	1	0.08	0.18	0.00	1.71	0.12	
	35-44 years old	30	0.36	2	0.17	0.23	0.02	1.44	0.12	
	45-54 years old	14	0.17	3	0.25	0.72	0.10	4.09	1.00	
Q6	Female	34	0.41	6	0.60	1.00	NA	NA	NA	FE
	Male	49	0.59	4	0.40	0.47	0.09	2.14	0.32	
Q7	College graduate (bachelor's degree)	51	0.61	5	0.42	1.00	NA	NA	NA	FE
	High school graduate or GED certificate	9	0.11	2	0.17	2.23	0.19	16.54	0.32	
	Post-graduate training or professional schooling after college	3	0.04	3	0.25	9.53	1.01	93.16	0.02	
	Trade school, associate's degree or some college	20	0.24	2	0.17	1.02	0.09	6.88	1.00	
Q37	More than 5 years	76	0.93	10	0.83	1.00	NA	NA	NA	FE
	Less than 5 years	6	0.07	2	0.17	2.50	0.22	16.76	0.27	

[a] The question (Q) number notations match those in the Qualtrics dataset.

[b] FE=Fisher's Exact Test

[c] In Q4, "Up to five years" included levels "Less than a year" and "Up to five years".

[d] In Q17, "Agree" includes "Strongly agree" and "Agree", while "Disagree" includes "Strongly disagree", "Disagree" and "Undecided".

[e] In Q10, "Interested" includes "I am already doing/have done this fully", "I would be ready to do this in the near future", and "I am interested but unable", while "Uninterested" includes "I am not interested in this".

[f] In Q15, "Agree" includes "Strongly agree", "Somewhat agree", and "Agree", while "Disagree" includes "Neither agree nor disagree", "Somewhat disagree".

[g] In Q18, "Agree" includes "Strongly agree and "Agree", while "Disagree" includes "Strongly disagree" and "Disagree".

[h] In Q5, "18-34 years old" includes "18-24 years old" and "25-34 years old".

Farm management survey

Survey Flow

Block: Default Question Block (40 Questions)

Page Break

Start of Block: Default Question Block

Q1

What is this survey?

You are invited to take part in a study about the management of dairy cattle in the Northeastern United States. We are seeking farm managers' experiences and opinions of farm management practices regarding the prevention and treatment of infectious diseases in cattle. This study is being conducted by the team of Dr. Renata Ivanek from the Department of Population Medicine and Diagnostic Sciences at Cornell University's College of Veterinary Medicine. This study is funded by a Federal Capacity Grant from the United States Department of Agriculture.

How long will it take?

The questionnaire will take about 20 minutes to complete.

Are there any risks?

We anticipate that your participation in this survey presents no greater risk than everyday use of the Internet. There are no costs to you for participating in the study. The information you provide will be analyzed and used to improve our understanding of the attitudes and opinions of dairy farmers regarding control of infectious diseases.

Are there any benefits?

The information collected may not benefit you directly, but the information learned in this study may help find solutions for managing infectious diseases in dairy cattle. As an incentive for participation, we are offering a $5 electronic gift card to Carhartt (an email address will need to be provided to receive gift card).

Will my answers be private and confidential?

This survey is anonymous. Do not write your name anywhere within the survey. No one will be able to identify you or your answers, and no one will know whether or not you participated in the study. Aside from your email address (only required for Carhartt gift card), no identifying information will be

collected. Therefore, your responses will remain anonymous. Any information that could be used to identify individuals that you might provide will remain confidential and will not be disclosed.

Is this voluntary?

Your participation in this study is voluntary. If you decide not to participate in this study, it will not affect your current or future relationship with Cornell University. By completing this survey, you are voluntarily agreeing to participate. You may choose to answer all or only a portion of the survey, however, we encourage you to answer as many questions as possible so as to increase the value of the information obtained from this study. If you decide to participate in this study, please complete the following electronic questionnaire.

Who do I contact if I have questions?

If you have any questions about the study, please contact veterinary student, Hannah Padda at hp396@cornell.edu. Alternatively, you may contact the primary researcher, Dr. Renata Ivanek, Associate Professor of Epidemiology, at Dept. of Population Medicine and Diagnostic Sciences, College of Veterinary Medicine, Cornell University, S1-072 Schurman Hall, Ithaca, NY 14853, Tel: 607-253-4383, Email: ri25@cornell.edu.

Has this project been approved by a review board?

The Cornell Institutional Review Board (IRB) has reviewed the request to conduct this project. If you have any concerns about your rights in this study, please contact the Cornell Institutional Review Board (IRB) at 607-255-5138, or you may access their website at http://www.irb.cornell.edu. You may also report your concerns or complaints anonymously through Ethicspoint (www.hotline.cornell.edu) or by calling toll free at 1-866-293-3077. Ethicspoint is an independent organization that serves as a liaison between the University and the person bringing the complaint so that anonymity can be ensured.

Thank you for your assistance in this important endeavor.

○ I have read the above description of this study and have been informed of the risks and benefits involved. (4)

Skip To: End of Survey If Q1 != I have read the above description of this study and have been informed of the risks and benefits involved.

Page Break

Q39 Tell us about your farm.

Q2 What most accurately describes your farm operation?

- ◯ Conventional (1)
- ◯ Organic (2)

Q3 What is your position/role on the dairy farm?

- ◯ Owner/co-owner (1)
- ◯ Herd manager (2)
- ◯ Other (3)

Skip To: End of Survey If Q3 = Other

Q4 How long have you owned or managed your farm?

- ◯ Less than a year (1)
- ◯ Up to five years (2)
- ◯ Between five and ten years (3)
- ◯ Between ten and twenty years (4)
- ◯ Over twenty years (5)

Page Break

Q40 Disease prevention practices.

Q8 How interested are you in adopting culture-based (pathogen based) testing for treating mastitis? Culture based testing involves taking a milk sample from a cow showing clinical mastitis in order to help identify the cause and best course of treatment.

- ◯ I am not interested in this (1)
- ◯ I am interested in this but unable (2)
- ◯ I would be ready to do this in the near future (3)
- ◯ I am already doing aspects of this (4)
- ◯ I am already doing this fully (5)

Display This Question:

If Q8 = I am interested in this but unable

Q9 If unable to adopt culture-based testing for treating mastitis, what is the primary barrier?

- ◯ Financial reasons (1)
- ◯ Lack of training in this area (2)
- ◯ Labor issues (3)
- ◯ No access to a lab for testing (4)
- ◯ Other (5) ____

Page Break

Q10 How interested are you in constructing a new barn or making similar significant changes to your farm facility to improve herd management?

- ◯ I am not interested in this (1)
- ◯ I am interested but unable (2)
- ◯ I would be ready to do this in the near future (3)
- ◯ I am already doing/have done this fully (4)

Display This Question:

If Q10 = I am interested but unable

Q11 If unable to construct a new barn or make similar significant changes to your farm facility, what is the primary barrier?

- ◯ Financial reasons (1)
- ◯ Labor issues (2)
- ◯ Local zoning regulations (3)
- ◯ Land not available (4)
- ◯ Other (5) ____

Page Break

Q42 Alternatives to antibiotics.

Q12 Which of the following product attributes is the most important if you needed to choose an antibiotic alternative to treat your lactating cows?

- ○ Ease of administration (1)
- ○ Scientifically proven (2)
- ○ Cost of the product (3)
- ○ Milk withhold time (4)
- ○ Allowed under organic certification (5)
- ○ Other (6) ____

Q13 Who or what sources do you consult the most often to learn about alternatives to antibiotics?

- ◯ Herd veterinarian (1)
- ◯ Cooperative Extension/Quality Milk Production Services (2)
- ◯ Dairy publications, such as Hoard's Dairyman (3)
- ◯ Nutritionist (4)
- ◯ Milk inspector (8)
- ◯ Other dairy farmers (7)
- ◯ None, I am not interested in antibiotic alternatives (5)
- ◯ Other (6) ____

Q14 How helpful is your veterinarian in advising you about alternatives to antibiotics?

- ◯ Not helpful (1)
- ◯ Somewhat helpful (2)
- ◯ Very helpful (3)
- ◯ Unsure (4)

Display This Question:

If Q14 = Not helpful

Q34 How could your veterinarian be more helpful in advising about alternatives to antibiotics?

____

Display This Question:

If Q14 = Somewhat helpful

Q35 What makes your veterinarian somewhat helpful in advising about alternatives to antibiotics?

____

Display This Question:

If Q14 = Very helpful

Q43 What makes your veterinarian very helpful in advising about alternatives to antibiotics?

____

Page Break

Q48 Optimizing antibiotic use.

Display This Question:

If Q2 = Conventional

Q15 State your level of agreement: "I would be interested in knowing how antibiotic use on my farm compares to use on other similar dairy farms in NY."

- Strongly agree (1)
- Agree (2)
- Somewhat agree (3)
- Neither agree nor disagree (4)
- Somewhat disagree (5)
- Disagree (6)
- Strongly disagree (7)

Display This Question:

If Q2 = Conventional

Q16 What primary external incentive would you need in order to reduce antibiotic use on your farm?

- ○ Financial incentives provided with your milk check (1)
- ○ Grants to upgrade facilities to reduce infection risk (2)
- ○ Subsidized veterinary consulting/Quality Milk Production Services (3)
- ○ Tax incentives (4)
- ○ Other (5) ____
- ○ None of the above (6)

Q17 Please state your level of agreement with the following statement: *"Antibiotic resistance due to antibiotic use in dairy farming may negatively impact the health of dairy cattle."*

- ○ Strongly agree (1)
- ○ Agree (2)
- ○ Undecided (3)
- ○ Disagree (4)
- ○ Strongly disagree (5)

Page Break

Q49 Mastitis treatment decisions.

Display This Question:

If Q2 = Conventional

Q18 State your level of agreement: "I treat with antibiotics at the first sign of mastitis infection."

- ○ Strongly disagree (1)
- ○ Disagree (2)
- ○ Agree (3)
- ○ Strongly agree (4)

Display This Question:

If Q18 = Strongly disagree

Or Q18 = Disagree

Q19 How important are each of the following when deciding to WAIT to give antibiotics after the first sign of mastitis?

	Extremely important (1)	Very important (2)	Moderately important (3)	Slightly important (4)	Not at all important (5)	Not applicable (6)
Cost of antibiotcs (1)	○	○	○	○	○	○
Concern about withholding violation (2)	○	○	○	○	○	○
Severity of health problem (3)	○	○	○	○	○	○
Lost revenue from withheld milk (4)	○	○	○	○	○	○
Goal of limiting antibiotic use (5)	○	○	○	○	○	○
Labor (how much help you have on the farm) (6)	○	○	○	○	○	○
Culture result (if applicable) (7)	○	○	○	○	○	○

Q21 How important are each of the following when deciding to give antibiotics after the first sign of mastitis?

	Extremely important (1)	Very important (2)	Moderately important (3)	Slightly important (4)	Not at all important (5)
Animal welfare (1)	○	○	○	○	○
Convenience of quickly solving the problem with antibiotic treatment (2)	○	○	○	○	○
Preventing disease spread (3)	○	○	○	○	○
Trust that treating sooner will have a higher chance of cure (4)	○	○	○	○	○

Display This Question:

If Q2 = Organic

Q22 If a cow could maintain its organic status, would you consider using antibiotics on your farm?

○ Definitely would not (1)

○ Probably would not (2)

○ Probably would (3)

○ Probably would not (4)

Page Break

Q47 On farm questions.

Q23 What was the average number of lactating cows in your herd for the last month?

____

Q31 How many cows did you dry off last month?

____

Q32 What was your average somatic cell count last month?

____

Q24 Over the past month, how many cases of clinical mastitis did you experience on your farm?

____

Q25 How many injectable antibiotic treatments have been provided to lactating cows on your farm in the last month? An antibiotic treatment is defined as a single dose, administered to a single animal. Please provide the total number of doses given across all lactating cows over the last month.

____

Q26 How many intramammary antibiotic treatments have been provided to lactating cows on your farm in the last month (not including dry cow treatment)? An antibiotic treatment is defined as a single dose,

administered to a single animal. Please provide the total number of doses given across all lactating cows over the past month.

____

Page Break

Q46 Demographic questions.

Q5 What is your age?

○ 18-24 years old (1)

○ 25-34 years old (2)

○ 35-44 years old (3)

○ 45-54 years old (4)

○ Over 55 (5)

Q6 What is your gender?

○ Male (1)

○ Female (2)

○ Other (3) ____

○ Prefer not to say (4)

Q7 What is the highest level of education you have completed?

- Eighth (8th) grade or less (1)
- Some high school (9-11) (2)
- High school graduate or GED certificate (3)
- Trade school, associate's degree or some college (4)
- College graduate (bachelor's degree) (5)
- Poster-graduate training or professional schooling after college (6)

Q37 How long do you plan to continue farming?

- Less than 5 years (1)
- More than 5 years (2)

Page Break

Q45 Thank you for your participation!

Q28 Please provide your email address for a $5 thank you gift card.

____

End of Block: Default Question Block
